# Robust Phagocyte Recruitment Controls the Opportunistic Fungal Pathogen *Mucor circinelloides* in Innate Granulomas *In Vivo*

**DOI:** 10.1128/mBio.02010-17

**Published:** 2018-03-27

**Authors:** Sarah Inglesfield, Aleksandra Jasiulewicz, Matthew Hopwood, James Tyrrell, George Youlden, Maria Mazon-Moya, Owain R. Millington, Serge Mostowy, Sara Jabbari, Kerstin Voelz

**Affiliations:** a Institute of Microbiology and Infection, University of Birmingham, Birmingham, United Kingdom; b School of Mathematics, University of Birmingham, Birmingham, United Kingdom; c School of Biosciences, University of Birmingham, Birmingham, United Kingdom; d Section of Microbiology, MRC Centre for Molecular Bacteriology and Infection, Imperial College London, London, United Kingdom; e Strathclyde Institute of Pharmacy & Biomedical Sciences, University of Strathclyde, Glasgow, United Kingdom; University of Melbourne

**Keywords:** fungal infection, fungal pathogenesis, host-pathogen interactions, innate immunity, macrophages, mathematical modeling, mucormycosis, neutrophils, phagocytes, zebrafish

## Abstract

Mucormycosis is an emerging fungal infection with extremely high mortality rates in patients with defects in their innate immune response, specifically in functions mediated through phagocytes. However, we currently have a limited understanding of the molecular and cellular interactions between these innate immune effectors and mucormycete spores during the early immune response. Here, the early events of innate immune recruitment in response to infection by *Mucor circinelloides* spores are modeled by a combined *in silico* modeling approach and real-time *in vivo* microscopy. Phagocytes are rapidly recruited to the site of infection in a zebrafish larval model of mucormycosis. This robust early recruitment protects from disease onset *in vivo*. *In silico* analysis identified that protection is dependent on the number of phagocytes at the infection site, but not the speed of recruitment. The mathematical model highlights the role of proinflammatory signals for phagocyte recruitment and the importance of inhibition of spore germination for protection from active fungal disease. These *in silico* data are supported by an *in vivo* lack of fungal spore killing and lack of reactive oxygen burst, which together result in latent fungal infection. During this latent stage of infection, spores are controlled in innate granulomas *in vivo*. Disease can be reactivated by immunosuppression. Together, these data represent the first *in vivo* real-time analysis of innate granuloma formation during the early stages of a fungal infection. The results highlight a potential latent stage during mucormycosis that should urgently be considered for clinical management of patients.

## IMPORTANCE

Mucormycosis is a dramatic fungal infection frequently leading to the death of patients. We know little about the immune response to the fungus causing this infection, although evidence points toward defects in early immune events after infection. Here, we dissect this early immune response to infectious fungal spores. We show that specialized white blood cells (phagocytes) rapidly respond to these spores and accumulate around the fungus. However, we demonstrate that the mechanisms that enable phagocytes to kill the fungus fail, allowing for survival of spores. Instead a cluster of phagocytes resembling an early granuloma is formed around spores to control the latent infection. This study is the first detailed analysis of early granuloma formation during a fungal infection highlighting a latent stage that needs to be considered for clinical management of patients.

## INTRODUCTION

Mucormycosis is the second most common fungal mold infection and is associated with extremely high mortality (up to >90% in disseminated infection) (1). Disease is caused by a spectrum of species belonging to the *Mucorales* (e.g., *Rhizopus oryzae*, *Mucor circinelloides*, and *Lichtheimia corymbifera*). Symptoms are due to spore germination and filamentous fungal growth within the host leading to angioinvasion, vessel thrombosis, and tissue necrosis (2–4). Although little epidemiological data is available for mucormycosis, estimates suggest approximately 10,000 infections are due to *Rhizopus oryzae* annually (5, 6). Individuals with hematopoietic disorders or transplants are particularly at risk of disease, with 8 and 16% of patients, respectively, presenting with mucormycosis (5). Mucormycosis is considered an emerging disease, with reported 7.3 and 9.3% increases in incidence and mortality between 2001 and 2010, respectively (7). Treatment of mucormycosis is very costly, with an average expense of $100,000 per case, and remains unsuccessful in most cases (8). Current antifungal therapy for mucormycosis is ineffective, and treatment involves extensive surgical removal of infected tissue, often leading to limb amputation and long-term disability. Therefore, there is clearly a clinical need for a more effective treatment strategy.

Mucorales are opportunistic pathogens. Thus, understanding how the immune system naturally prevents pathological disease and identifying the crucial components may inform future targets for mucormycosis therapy. Susceptible patients often present with innate immune defects (for example, neutropenia and impaired macrophage or neutrophil functions) due to uncontrolled diabetes or corticosteroid therapy (1, 5, 9, 10). This highlights the essential role of phagocytes for an effective immune response to mucormycosis. To counteract the immune response, there is strong evidence for an immune-inhibitory effect of infectious fungal spores (11, 12).

Traditional animal models to study the pathogenesis of mucormycosis have been limited and do not allow for the integrative study of cellular interactions (13). Host expression studies of *R. oryzae* infections in the fruit fly (*Drosophila melanogaster*) infection model showed downregulation of genes in several pathways, including pathogen recognition, immune defense, and stress responses (14). Murine bronchoalveolar macrophages inhibit germination of *R. oryzae* spores and thus prevent formation of invasive hyphal forms in the healthy host (11, 12, 15, 16). However, macrophages and neutrophils are unable to kill fungal spores (11, 12, 17). In addition, macrophages from diabetic or corticosteroid-treated mice fail to inhibit spore germination (15, 18). Despite this, the molecular and cellular interactions during the early immune response to mucormycete spores are poorly understood. Yet, this information may offer an immunomodulatory target for protection from disease onset. Using our *in vivo* larval zebrafish (*Danio rerio*) model of mucormycosis, we previously demonstrated that macrophages and neutrophils are rapidly recruited to the site of infection with *M. circinelloides* (17).

In this study, we model the early events of innate immune recruitment in response to *M. circinelloides* spores by a combined *in silico* modeling and real-time microscopy approach *in vivo*. Utilizing our recently established zebrafish infection model (17), we took advantage of the optical transparency of zebrafish larvae, which allow for real-time microscopy, to model the early temporal-spatial events of innate immune recruitment in response to fungal spores *in vivo*. We combined this experimental analysis with mathematical modeling to identify the essential parameters for protection from pathological disease. We showed that robust early phagocyte recruitment is crucial for protection from disease. Proinflammatory cytokine signals (e.g., early transcription response of the *tnf*-α and and *il-8* genes, coding for tumor necrosis factor alpha [TNF-α] and interleukin-8 [IL-8], respectively) are important for induction of effective phagocyte recruitment. Yet, our data suggest that protection is dependent on the capacity, rather than the rate, of phagocyte recruitment to the site of infection. At the site of infection, phagocytes form tight clusters around spores resembling early granulomas. While spores are successfully contained in these early granulomas, which we term innate granulomas, we observe a lack of reactive oxygen burst and failure to kill fungal spores. Moreover, disease can be reactivated by dexamethasone (Dex)-induced immunosuppression. Together, this is the first real-time analysis of innate granuloma formation during the early stages of a fungal infection. Our data indicate the potential for a latent infectious stage during mucormycosis that needs to be considered for clinical management of patients.

## RESULTS

### Phagocytes are rapidly recruited to the site of *Mucor circinelloides* infection *in vivo.*

**FIG 1:**
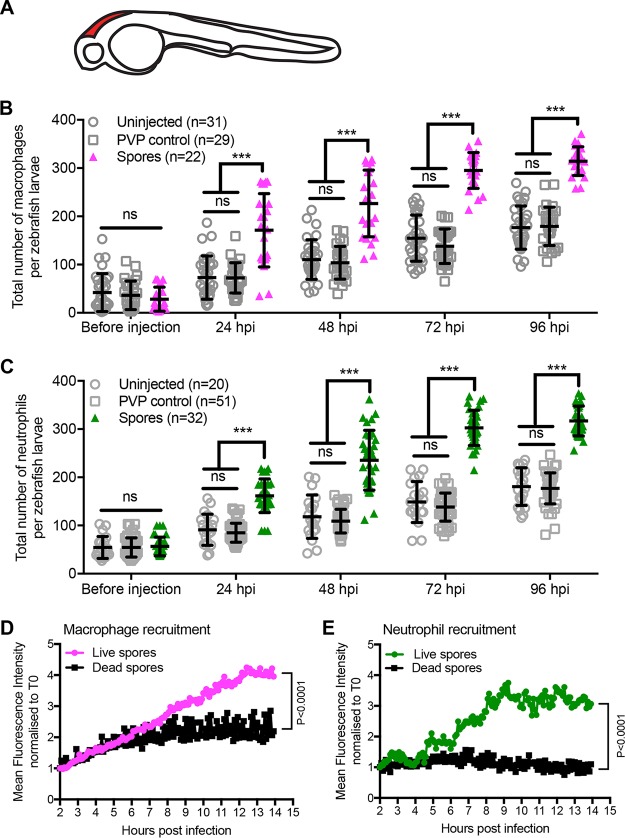
Phagocytes are rapidly recruited to the site of infection with live *Mucor circinelloides in vivo*. (A) Schematic of a prim-25 zebrafish embryo indicating the site of infection (the hindbrain ventricle) in red. (B and C) Macrophage and neutrophil total body numbers were monitored in Tg(mpeg1:G/U:NfsB-mCherry) and Tg(mpx:GFP) zebrafish larvae, respectively, in uninjected larvae, or after injection of control medium (PVP) or 100 spores into the hindbrain of 36-hpf larvae. Phagocyte numbers were counted before microinjection and every 24 h after injection. Both macrophage and neutrophil numbers increase with larval age, while spore exposure significantly increases the total number of macrophages (B) and neutrophils (C). Individual data points and the mean average and standard error of the mean are presented. Statistical analysis was performed on data using two-way ANOVA with Tukey’s multiple-comparison test (***, *P* < 0.001; ns, not significant). (D and E) Macrophage and neutrophil recruitment in response to *Mucor circinelloides* was monitored by real-time microscopy over 12 h, starting 2 h postinfection in transgenic zebrafish Tg(mpeg1:G/U:NfsB-mCherry/mpx:GFP) larvae (17), and recruitment was assessed by mean fluorescence intensity measurements using Volocity image analysis software. Macrophages (D) and neutrophils (E) were rapidly recruited to the site of infection of live spores. Recruitment was significantly reduced in response to dead spores (*P* < 0.0001, analysis of covariance).

Zebrafish larvae do not have an adaptive immune system and rely on their innate immune cells to respond to infectious stimuli. We took advantage of this characteristic to define the phagocyte response to infection with *M. circinelloides* asexual spores. We used transgenic zebrafish larvae with fluorescently tagged macrophages [Tg(mpeg1:G/U:NfsB-mCherry)] and neutrophils [Tg(mpx:GFP)] to assess whole-larval phagocyte number. Phagocyte recruitment was observed by fluorescence microscopy in uninjected larvae or after microinjection of control medium (polyvinylpyrrolidone [PVP]) or 100 *M. circinelloides* spores in the hindbrain ventricle of 36-h-old zebrafish larvae (Fig. 1A). Phagocyte numbers were counted before microinjection and every 24 h after injection. Both macrophage and neutrophil numbers increased with larval age, even in untreated larvae (Fig. 1B and C). However, the total numbers of macrophages (*P* < 0.001, two-way analysis of variance [ANOVA]) and neutrophils (*P* < 0.001, two-way ANOVA) in the whole larvae increased 11.2- and 5.6-fold, respectively, following spore injection compared to 5.0- and 3.2-fold increases, respectively, in control-injected larvae (Fig. 1B and C). This suggested a global innate immune response to *M. circinelloides* infection in this model system.

Next, we wanted to understand how this global increase in phagocyte numbers correlates to phagocyte recruitment to the site of infection. We previously conducted real-time imaging to qualitatively illustrate that phagocytes are recruited in response to *M. circinelloides* spores in our *in vivo* zebrafish larval hindbrain ventricle model (17). Here, we analyzed these movies quantitatively by high-resolution tracking of phagocyte movement from the whole fish toward infectious fungal spores at the site of infection using the software Volocity. Data were analyzed for the first 14 h postinfection (hpi). Mean fluorescence intensity (MFI) for mCherry-tagged macrophages and green fluorescent protein (GFP)-tagged neutrophils was measured at the site of infection in transgenic zebrafish larvae. Infected larvae exhibit rapid onset of macrophage recruitment to the site of infection with live spores. The number of macrophages steadily increased until maximum recruitment was achieved 12 hpi (Fig. 1D). Macrophage recruitment in response to infection with dead spores was 2.1-fold lower (*P* < 0.0001, analysis of covariance) (Fig. 1D). Onset of neutrophil recruitment was delayed until 4 hpi, after which neutrophil recruitment proceeded until 9 hpi, when maximum recruitment was reached (Fig. 1E). There were 3.0-fold fewer neutrophils recruited to the site of infection in response to dead spores compared to injection with live spores (*P* < 0.0001, analysis of covariance) (Fig. 1E).

Collectively, these data show that zebrafish respond rapidly to infection with live *M. circinelloides* spores both by increasing their phagocyte number and by recruiting phagocytes to the site of infection.

### Robust early phagocyte recruitment protects from *Mucor circinelloides in vivo.*

Immunosuppression predisposes individuals to mucormycosis. However, the mechanism for this enhanced susceptibility is not fully understood. We employed dexamethasone (40 μg/ml), a well-established corticosteroid that mimics immune inhibition in the context of mucormycosis (17, 19, 20), to analyze the impact on phagocyte recruitment after infection with mucormycete spores. Our zebrafish model lacks a fully functional adaptive immune system. In this context, dexamethasone’s immune-inhibitory effects would most likely affect the innate immune response by inhibiting proinflammatory mechanisms.

**FIG 2:**
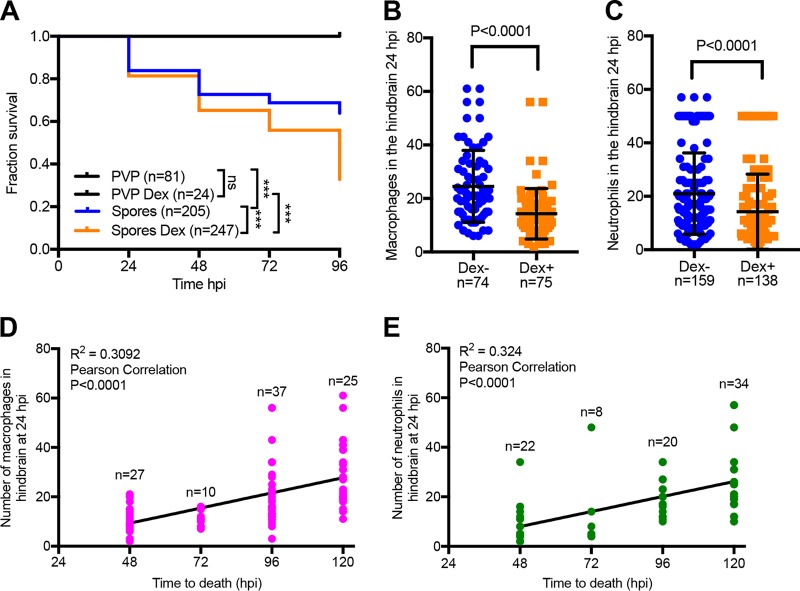
Robust phagocyte recruitment protects from *Mucor circinelloides in vivo*. General immunosuppression was induced in zebrafish larvae with the steroid dexamethasone (40 μg/ml), and zebrafish were monitored for mortality and phagocyte recruitment. (A) Dexamethasone treatment significantly increased larval mortality after infection with fungal spores (*P* < 0.001, Mantel-Cox log-rank test) while significantly reducing the number of macrophages and neutrophils recruited to the hindbrain ventricle (site of infection) in Tg(mpeg1:G/U:NfsB-mCherry) (B) and Tg(mpx:GFP) (C) embryos, respectively, 24 hpi (*P* < 0.0001, Mann-Whitney *U* test). (D and E) The number of macrophages and neutrophils at the site of infection at 24 hpi was significantly correlated with the time of observed death of infected zebrafish (*P* < 0.0001, Pearson correlation). A higher phagocyte number in the hindbrain ventricle at 24 hpi resulted in longer larval survival after infection.

Dexamethasone-treated larvae infected in the hindbrain ventricle exhibited significantly increased mortality relative to untreated larvae (67.2% versus 36.1%; *P* < 0.0001, Mantel-Cox log-rank test) (Fig. 2A). This difference is most visible 96 hpi, mostly likely due the invasive fungal growth having reached the critical threshold leading to host death. At the same time, dexamethasone treatment significantly reduced the numbers of macrophages (1.7-fold; *P* < 0.0001, Mann-Whitney *U* test) and neutrophils (1.5-fold; *P* < 0.0001, Mann-Whitney *U* test) recruited to the site of infection 24 hpi (Fig. 2B and C). We followed the fate of these larvae and recorded the time of larval death. There was a strong correlation between the numbers of macrophages (*P* < 0.0001, Pearson correlation) and neutrophils (*P* < 0.0001, Pearson correlation) and the time of mortality (Fig. 2D and E). While macrophage and neutrophil numbers were significantly reduced at the site of infection after dexamethasone treatment, the corticosteroid did not alter overall phagocyte numbers (whole body) or inhibit the global increase in phagocyte numbers in response to the infectious stimulus (see Fig. S1 in the supplemental material). Whole-larval phagocytes also outnumbered recruited phagocytes, indicating that recruitment was not limited by phagocyte availability (Fig. 1B and C and 2B and C; Fig. S1).

10.1128/mBio.02010-17.1 FIG S1  Dexamethasone treatment does not impact phagocyte numbers. We counted total macrophage and neutrophil numbers with and without dexamethasone intervention to ensure that dexamethasone treatment did not change the number of phagocytes available for recruitment to the site of infection (A and B). We observed a significant increase in whole-zebrafish macrophage (A) and neutrophil (B) numbers at 24 hpi compared to 36 hpf with (Dex+) and without (Dex−) dexamethasone treatment (*P* < 0.001, Kruskal-Wallis test with Dunn’s multiple-comparison test). At the same time, dexamethasone did not significantly alter the number of macrophages (A) or neutrophils (B) in the whole larvae at 24 hpi (ns, not significant, Kruskal-Wallis test with Dunn’s multiple-comparison test). Download FIG S1, PDF file, 0.1 MB. Copyright © 2018 Inglesfield et al. 2018 Inglesfield et al. This content is distributed under the terms of the Creative Commons Attribution 4.0 International license.

Taken together, these data suggest that phagocyte recruitment to the site of infection is an essential aspect in the prevention of morbidity and mortality. Moreover, phagocyte numbers recruited to the site of infection 24 hpi may have predictive value for disease progression and/or outcome.

### The importance of phagocyte recruitment for protection from *Mucor circinelloides* can be modeled by an *in silico* approach.

**FIG 3:**
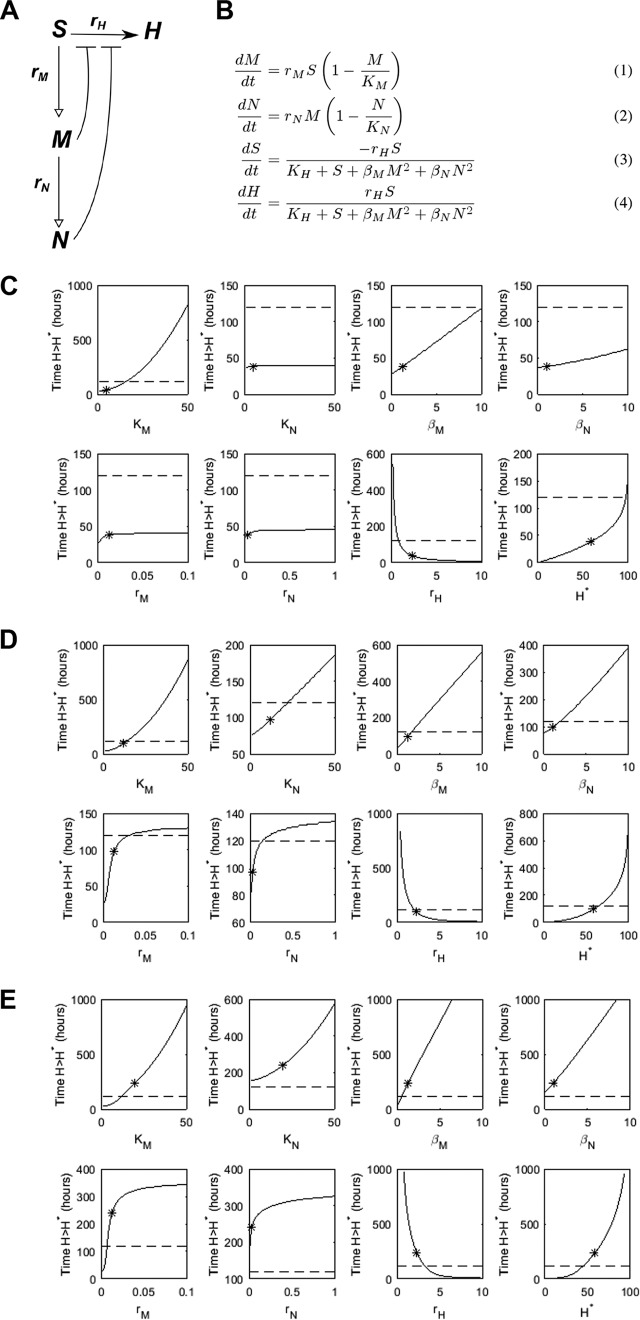
Mathematical model of the innate immune response to a mucormycosis infection. A schematic representation is given in panel A, where open-headed arrows illustrate recruitment, solid arrows germination, and bars inhibition. Model equations are given in panel B. Phagocytes are assumed to have a carrying capacity at which they will saturate. Germination is inhibited by the presence of phagocytes. Variables: *S*, spores; *H*, hyphae; *M*, macrophages; *N*, neutrophils. Parameters: *r*_*M*_/*r*_*N*_, macrophage/neutrophil recruitment rates; *K*_*M*_/*K*_*N*_, macrophage/neutrophil carrying capacities (maximum number at infection site); *r*_*H*_, spore germination rate; *K*_*H*_, germination saturation constant; β_*M*_/β_*N*_, macrophage/neutrophil germination inhibition coefficients. (C to E) Individual parameter variations to the mathematical model using three categories of zebrafish: “immunocompromised” (C), “susceptible” (D), and “healthy” (E). Shown is the impact of variation of individual parameters (indicated by *x* label) on time taken to reach H* (indicating death from mucormycosis). The dashed line marks 120 h: where the solid line lies above the dashed line, zebrafish are deemed to survive for corresponding parameter values. The asterisk indicates the default parameter value (i.e., prior to variation). The horizontal distance between the asterisk and the point at which the solid curve crosses the dashed line indicates the required absolute change to the parameter to support survival.

Our experimental data demonstrate that whole-fish phagocyte count does not predict outcome. Rather, local phagocyte numbers at the site of infection affect infection outcome. To investigate this further, we took a mathematical modeling approach to perturb parameters affecting the system of interest, either individually or in combination. Such an investigation is relatively straightforward compared to its experimental equivalent and can yield vital insight into a system and help to guide future experimental work. We therefore modeled infection dynamics using the schematic presented in Fig. 3A and B. We thus present results for this model based on three categories of zebrafish, each distinguished by their maximum phagocyte number at the infection site (carrying capacity): an “immunocompromised” zebrafish (maximum macrophage number *K*_*M*_ = 5, maximum neutrophil number *K*_*N*_ = 5), a “susceptible” zebrafish (*K*_*M*_ = *K*_*N*_ = 12), and a “healthy” zebrafish (*K*_*M*_ = *K*_*N*_ = 20). Our experimental observations showed that if an infected zebrafish survived until after 96 h postinfection (here denoted as 120-h survival), it was likely to survive the infection entirely. We thus demark 120 h as a survival point if the number of hyphae has not yet passed the critical threshold, H*.

We simulated variations in all individual model parameters (Fig. 3A and B) (within what might be considered a reasonable range) bar *K*_*H*_ (which was not deemed to be an aspect of the system that could be manipulated in practice), while fixing the other parameters at their default values (with phagocyte carrying capacities as given above) and recorded the change in predicted survival time (Fig. 3C to E). In agreement with the experimental findings that phagocyte number at the site of infection (carrying capacity) is crucial in influencing disease outcome, the ability to support survival by varying any one of the parameters was highly dependent upon the maximum number of phagocytes at the infection site. For the “immunocompromised” zebrafish (low *K*_*M*_, *K*_*N*_), only a decrease in *r*_*H*_ (germination rate) or a large increase in *K*_*M*_ (macrophage carrying capacity) changed survival outcome (Fig. 3C). In contrast, survival could be supported in the “susceptible” zebrafish (intermediate *K*_*M*_, *K*_*N*_) by varying any individual parameter within the range tested (Fig. 3D). Notably, in these zebrafish simulations, relatively small increases to parameters associated with the macrophage response (particularly *K*_*M*_) were sufficient to support survival. While altering the phagocyte recruitment rates (*r*_*M*_, *r*_*N*_) could also support survival, a large fold increase was required for these parameters: i.e., the effectiveness of the immune response may be more sensitive to changes in the maximum number of macrophages present at the infection site than it is to the speed at which these immune cells can arrive at the infection site. The “healthy” zebrafish (large *K*_*M*_, *K*_*N*_) could, to an extent, cope with worsened parameter values (e.g., *K*_*N*_ = 0) and still survive (Fig. 3E).

Thus, our mathematical model can predict our experimental data that sufficient phagocyte recruitment to the site of infection is essential for protection from mucormycosis.

### *In silico* modeling indicates that protection from *Mucor circinelloides* is dependent on maximum phagocyte number at the site of infection.

**FIG 4:**
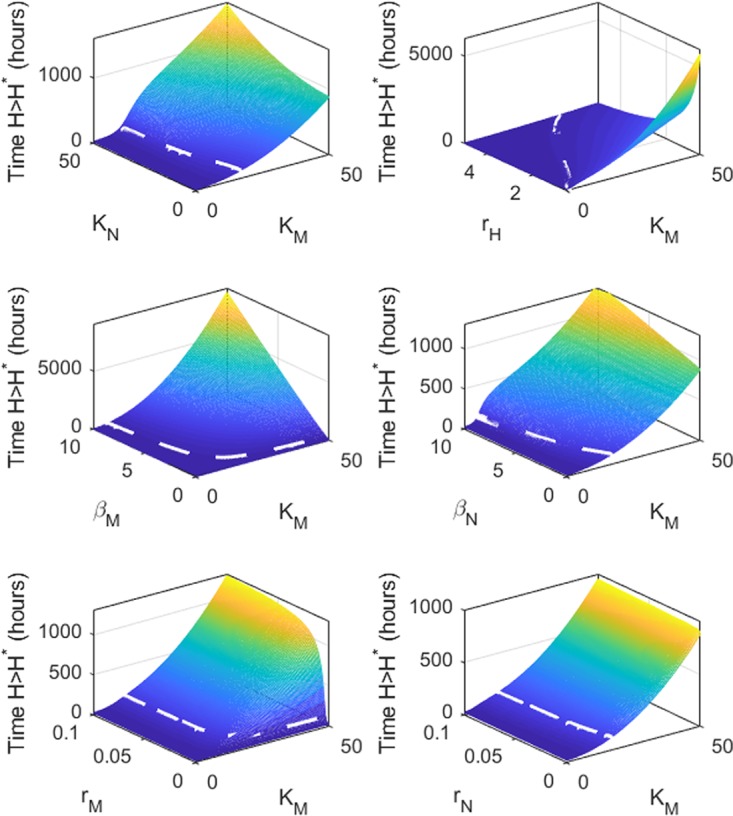
Double-parameter variations indicate that protection from *Mucor circinelloides* is dependent on maximum phagocyte number at the site of infection (varying *K*_*M*_ and an additional parameter as indicated by the *y* label) as applied to the mathematical model using “susceptible” zebrafish parameters (*K*_*N*_ = 12), and impact of their variation on time taken to reach H* (indicating death from mucormycosis). The dashed line marks 120 h: where the plane lies above the dashed white line, zebrafish are deemed to survive for the corresponding parameter values.

The variation between our three categories of zebrafish suggests that maximum phagocyte numbers at the infection site (particularly *K*_*M*_) are not only a critical factor to survival, but are capable of influencing the effect of varying the other parameters on survival time: i.e., the sensitivity of the immune response to the other parameters varies depending on the value of *K*_*M*_. To examine this phenomenon further, we varied *K*_*M*_ and each other parameter simultaneously and examined the impact on survival time. We illustrate only those results for the “susceptible” zebrafish (Fig. 4). Most striking is that, for survival, some parameters are dispensable with a sufficiently high *K*_*M*_. There were minimum thresholds required for those parameters associated with macrophage function: i.e., nonzero *r*_*M*_ and β_*M*_ are needed for survival. (The first of these is intuitive since macrophage arrival at the infection site is also required for the secondary neutrophil response.) By varying *r*_*M*_ and *K*_*M*_ simultaneously, we see that having a small number of macrophages arrive quickly at the infection site is likely to be less influential than having large numbers arrive more slowly. For sufficiently high *r*_*H*_, death was always predicted before 120 h regardless of *K*_*M*_ (in the range tested). Since macrophages are assumed to be required for neutrophil recruitment, in no case could the infection be survived if *K*_*M*_ = 0. Given the dominance of *K*_*M*_ in these simulations, equivalent results for the “immunocompromised” zebrafish are highly similar despite a lower *K*_*N*_ value (see Fig. S2 in the supplemental material).

10.1128/mBio.02010-17.2 FIG S2  Double-parameter variations indicate that protection from *Mucor circinelloides* is dependent on maximum phagocyte number at the site of infection (varying *K*_*M*_ and an additional parameter as indicated by the *y* label) in the mathematical model using “immunocompromised” zebrafish parameters (*K*_*N*_ = 5), and the impact of their variation on time taken to reach H* (indicating death from mucormycosis). The dashed line marks 120 h: where the plane lies above the dashed white line, zebrafish are deemed to survive for the corresponding parameter values. Download FIG S2, PDF file, 0.2 MB. Copyright © 2018 Inglesfield et al. 2018 Inglesfield et al. This content is distributed under the terms of the Creative Commons Attribution 4.0 International license.

Taken together, our mathematical model highlights the maximum number of phagocytes at the site of infection as the critical factor for host survival.

### *In silico* modeling indicates the role of proinflammatory signals for phagocyte recruitment and highlights the role of inhibition of spore germination for protection from active mucormycosis.

**FIG 5:**
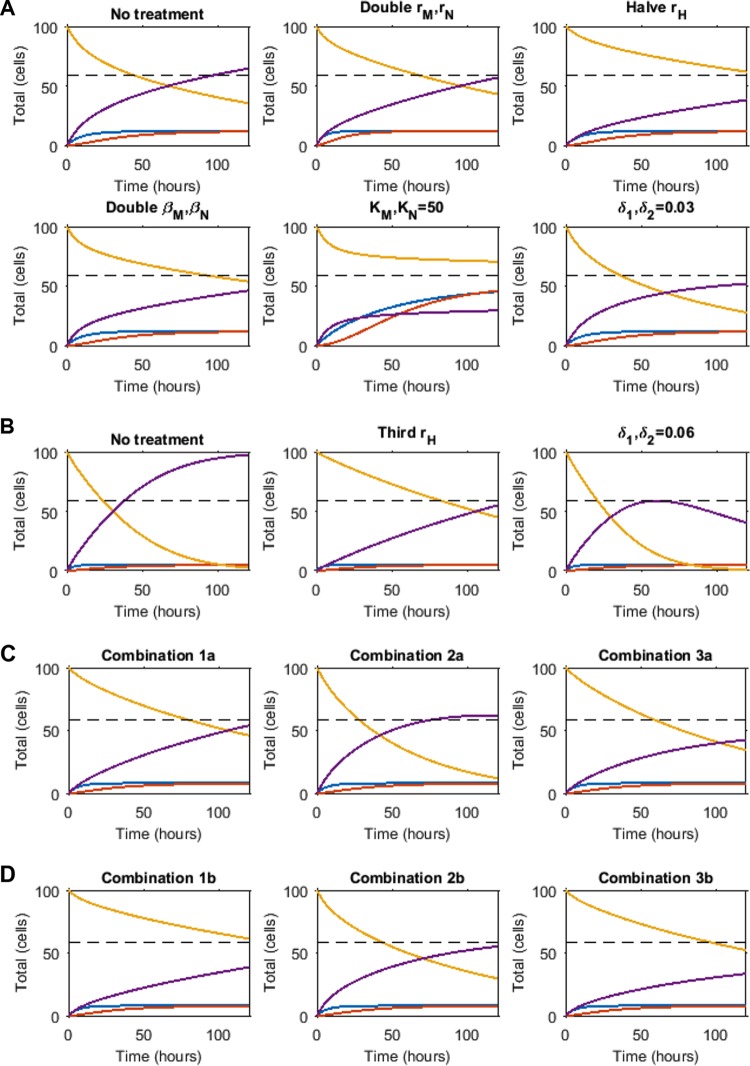
Simulation of protective mechanisms from onset of mucormycosis. Simulations of zebrafish with (A) *K*_*M*_ = *K*_*N*_ = 12 and (B) *K*_*M*_ = *K*_*N*_ = 5 were run with a proinflammatory response induced as indicated above the individual plots. The responses mimicked were inducing phagocyte recruitment (*r*_*M*_ and *r*_*N*_), or function (β_*M*_ and β_*N*_), inhibiting hyphae formation (*r*_*H*_), increasing maximum phagocyte number (*K*_*M*_, *K*_*N*_), and phagocyte-induced spore/hyphae death (δ_1_, δ_2_). For the last of these, equations 3 and 4, respectively, from Fig. 3B are altered to become
$$\frac{dS}{dt}=\frac{-r_{H}S-\delta_{1}S(M+N)}{K_{H}+S+\beta_{M}M^{2}+\beta_{N}N^{2}}$$
and
$$\frac{dH}{dt}=\frac{r_{H}S-\delta_{2}H(M+N)}{K_{H}+S+\beta_{M}M^{2}+\beta_{N}N^{2}}$$
For a proinflammatory response to facilitate survival, the purple line must not cross the dashed line during the time course. (C and D) Simulation of combinations of improved phagocyte carrying capacity and functionality for “immunocompromised” zebrafish. Simulations mimic an increase in phagocyte carrying capacity to *K*_*M*_, *K*_*N*_ = 8 in panel C and with an additional increase in phagocyte function in panel D (β_*M*_, β_*N*_ doubled). In combination 1, the hyphal formation rate, *r*_*H*_, is halved. In combination 2, a low level of phagocyte-induced killing of hyphae and spores is simulated (δ_1_, δ_2_ = 0.03). In combination 3, both of these additional responses are induced. Each population is illustrated: hyphae in purple, spores in yellow, macrophages in blue, and neutrophils in red. The dashed line marks H* (the threshold number of hyphae above which zebrafish are assumed to die). Survival is predicted if the number of hyphae remains below H* during the time course simulated.

Together, our experimental and *in silico* data suggest that the presence of phagocytic cells at the site of infection, rather than the rate at which the pool of available phagocytic cells arrive, is a crucial determinant of disease outcome. Proinflammatory cytokines are well-known stimuli that sequester the recruitment of immune cells. Patients with reduced proinflammatory capacity due to medical interventions such as corticosteroid treatment have higher risk of mucormycosis (3, 5, 21). We thus assessed the impact of proinflammatory cytokine expression on effective phagocyte recruitment. We modeled a series of time-dependent simulations representing various proinflammatory responses and examined their effects on mucormycosis outcome. We tested the impact of the rates of phagocyte recruitment (*r*_*M*_ and *r*_*N*_), the inhibitory function of phagocytes (β_*M*_ and β_*N*_), the carrying capacities of the phagocytes (*K*_*M*_ and *K*_*N*_), and the rate of fungal germination (*r*_*H*_). Assuming there would be a limitation on how much these rates could be altered in reality, relatively small changes to parameters were used, doubling or halving as appropriate, with the exception of *K*_*M*_ and *K*_*N*_, which were allowed to vary up to a total number of 50 (as observed in experiments). We also mimicked a proinflammatory response, whereby *in silico* phagocytes could directly clear spores and hyphae (see Fig. 5 for details of the model alteration). The above responses were applied to the parameter sets for “susceptible” (*K*_*M*_ = *K*_*N*_ = 12) and “immunocompromised” (*K*_*M*_ = *K*_*N*_ = 5) zebrafish, with an aim to facilitate survival (defined to occur if *H* < H* for all times *t* ≤ 120 h). Two different profiles were required to protect either the “immunocompromised” or the “susceptible” zebrafish. First, the “susceptible” zebrafish was receptive to a variety of perturbations (Fig. 5A). The most efficacious strategies to protect from onset of active mucormycosis were by directly inhibiting the rate of hyphae formation (*r*_*H*_), doubling the ability of immune cells to inhibit hyphae formation (*β*_*N*_, *β*_*M*_), or increasing the numbers of macrophages (*K*_*M*_) and neutrophils (*K*_*N*_). The last of these, which provides the zebrafish with more phagocytes or neutrophils at the infection site than the previously defined baseline “healthy” zebrafish, achieved the lowest hyphae number over the 120-h time course, in line with the importance of phagocyte carrying capacity in protecting from disease. An increase to only *K*_*M*_, *K*_*N*_ = 14 (i.e., fewer than the “healthy” zebrafish) also predicted survival until 120 h, but with formation of more hyphae (results not shown), indicating a threshold number for partial control. In addition, direct killing of spores and hyphae by phagocytes was predicted as a successful strategy to support survival, with δ_1_, δ_2_ = 0.03 (where δ_1_ and δ_2_ are the maximum rates of spore and hyphae killing, respectively). In contrast, the three most effective strategies for the “susceptible” zebrafish were the only three modifications that conferred protection on the “immunocompromised” zebrafish: an increase in maximum phagocyte number at the infection site (equivalent simulation to the “susceptible” zebrafish); direct killing of spores and hyphae by phagocytes, although this needed to be at a 2-fold higher rate than for the “susceptible” zebrafish (δ_1_, δ_2_ = 0.06); or a 3-fold decrease in the germination rate (*r*_*H*_) (as opposed to a 2-fold decrease for the “susceptible” zebrafish) (Fig. 5B).

Given the fast progression of mucormycosis and rapid hyphal growth under immunosuppressed conditions, we tested the relative contributions of phagocyte carrying capacity and inhibition of spore germination to disease establishment. Two scenarios were used: first, macrophage carrying capacities were increased by a relatively small amount to *K*_*M*_, *K*_*N*_ = 8 (Fig. 5C); second, in addition to this increase in carrying capacity, macrophage function (germination inhibition coefficients β_*M*_ and β_*N*_) was doubled (Fig. 5D). Neither of these approaches in isolation was capable of ensuring the zebrafish survive the infection under this parameter set. We then combined both scenarios with alterations to the following responses postinfection: (i) halving the hyphae formation rate, *r*_*H*_; (ii) phagocyte-induced killing of hyphae and spores introduced (δ_1_, δ_2_ = 0.03); or (iii) both of the preceding. Inhibition of hyphae formation in combination with the increased macrophage carrying capacity was protective against mucormycosis (Fig. 5C, combination 1a), while phagocytic killing at the simulated rate did not sufficiently inhibit hyphal growth, even at these enhanced macrophage levels (Fig. 5C, combination 2a). Under our parameter set, an increase in phagocytic killing is only protective under increased germination-inhibiting potential (Fig. 5C, combination 3a). Increasing both phagocyte numbers and function (i.e., germination inhibition coefficients) proved to be the most effective at restricting hyphal number (compare Fig. 5C and D).

Together, these *in silico* data suggest that a single proinflammatory response alone (i.e., phagocytic recruitment or killing) may be insufficient to prevent hyphal growth and protect against fulminant mucormycosis in immunocompromised hosts.

### Phagocytes control *Mucor circinelloides* spores in innate granulomas *in vivo.*

Our model indicates several protective mechanisms that would support mucormycosis management. We therefore tested a range of these predictions.

**FIG 6:**
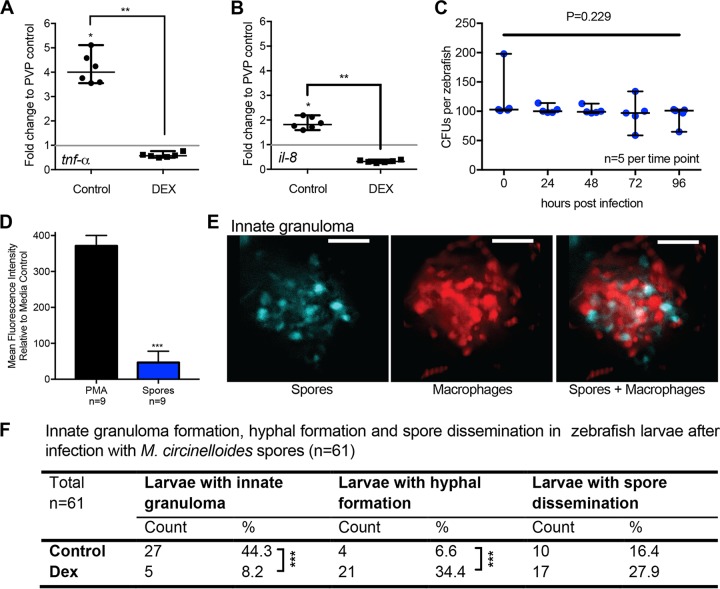
Phagocytes control *Mucor circinelloides* spores in innate granulomas *in vivo*. (A and B) We tested the mathematical prediction that proinflammatory cytokine expression supports phagocyte recruitment by evaluating the early transcription response (1 hpi) of two proinflammatory cytokine genes (*tnf*-α and *il-8*, coding for tumor necrosis factor alpha and interleukin-8, respectively) after spore exposure in control- and dexamethasone-treated fish. Dexamethasone-treated fish showed significantly lower levels of transcript for both cytokines (*P* < 0.01 [*n =* 6 with 15 fish each], Mann-Whitney *U* test). (C) While phagocyte-induced spore death was identified by our *in silico* modeling as an efficient mechanism to protect from onset of mucormycosis, and we showed rapid and robust recruitment of phagocytes to the site of infection *in vivo*, spores are not cleared from zebrafish larvae over a time course of 96 h after infection, as demonstrated by CFU countings indicating live spores (*P* = 0.229 [*n =* 5], Kruskal-Wallis test). (D) We were able to show a lack of induction of reactive oxygen species (ROS) in response to fungal spores compared to the ROS-inducing stimulus PMA (*P* < 0.0001 [*n =* 9], Mann-Whitney *U* test), indicating a lack of antimicrobial activity. (E) Representative image of an innate granuloma formed of fungal spores and macrophages (maximum-intensity projection of fluorescent z-stack with 43 sections every 4 μm; scale bar, 100 μm). (F) Dexamethasone treatment decreases the formation of innate granulomas (*P* < 0.0001 [*n =* 61], Fisher’s exact test) while increasing hyphal formation (*P* < 0.0001 [*n =* 61], Fisher’s exact test) and the occurrence of spore dissemination.

First, our model indicates that proinflammatory signals are essential for the recruitment of phagocytes. Thus, we here assessed the early (1 hpi) transcriptional response of the proinflammatory cytokine gene *tnf*-α and the chemotactic cytokine gene *il-8* in control- and dexamethasone-treated fish. We observed a significant transcriptional upregulation for both cytokines (*tnf*-α, 4.1-fold; *il-8*, 1.9-fold) in spore-injected larvae compared to mock-injected (PVP) larvae (*P* < 0.05 [*n =* 6], Mann-Whitney *U* test) (Fig. 6A and B). After dexamethasone treatment, the transcriptional response for both proinflammatory cytokines after spore injection was reduced compared to mock-injected fish (*tnf*-α, 0.6-fold; *il-8*, 0.3-fold) and significantly lower than in untreated fish (Fig. 6A and B) (*P* < 0.01 [*n =* 6], Mann-Whitney *U* test). These data are in agreement with our mathematical modeling results.

Second, our model indicates spore killing by phagocytes would be an effective protective response to infection. However, we had previously shown that spores remain viable inside the zebrafish for 48 hpi, despite rapid and robust recruitment of phagocytes to the site of infection (17). We now extended our assessment of spore viability to 96 hpi, demonstrating that there is no reduction in spore viability upon infection, even after contact with phagocytes for 96 h (Fig. 6C) (*P* = 0.229 [*n =* 5], Kruskal-Wallis test). This indicates a lack of antimicrobial activity by these recruited phagocytes. The induction of reactive oxygen species (ROS) is a well-known phagocytic mechanism to achieve microbial killing (15, 22–25). Thus, we measured the ROS production in response to fungal spores in the whole zebrafish embryo. In comparison to an ROS-inducing stimulus that generates ROS levels similar to those enabling *Candida albicans* killing by neutrophils (26), infection with spores stimulated significantly lower ROS levels (*P* < 0.0001 [*n =* 9], Mann-Whitney *U* test) (Fig. 6D).

Together, these data raise the question as to how *M. circinelloides* is controlled in zebrafish larvae. We have previously demonstrated that, upon infection, a dense phagocyte cluster forms around the spores (17) (Fig. 6E). We have termed this cluster an innate granuloma. Similar innate granulomas have been described in the zebrafish larval model for *Mycobacterium tuberculosis*, a bacterium that is contained in granulomas (27). Our *in silico* data predict that innate granuloma formation, which localizes phagocytic cells to the site of infection, is important for disease control. Therefore, to test *in vivo* if the formation of innate granulomas in response to infection with *M. circinelloides* spores is required to control the fungal infection, we immunosuppressed larvae with dexamethasone and monitored innate granuloma formation, hyphal formation, and spore dissemination. The formation of innate granulomas was significantly reduced in immunosuppressed zebrafish (*P* < 0.0001 [*n =* 61], Fisher’s exact test) (Fig. 6F), while at the same time hyphal formation was significantly increased (*P* < 0.0001 [*n =* 61], Fisher’s exact test) (Fig. 6F), and there was an overall trend of increasing spore dissemination (Fig. 6F).

In summary, our data demonstrate the importance of phagocyte carrying capacity at the site of infection (Fig. 3 and 4), a lack of spore killing (Fig. 6C), and an increase of larval mortality with lack of phagocyte recruitment to the site of infection (Fig. 2). Together with our previously reported data demonstrating the lack of phagocyte cluster formation after injection with dead spores (17), we propose a model in which *M. circinelloides* spores establish latent infections in the host. The host in turn contains the spores in innate granulomas, a mechanism to achieve full carrying capacity at the site of infection, to control the fungal infection.

### Latent infection with *Mucor circinelloides* can be activated out of innate granulomas by immunosuppression *in vivo.*

**FIG 7:**
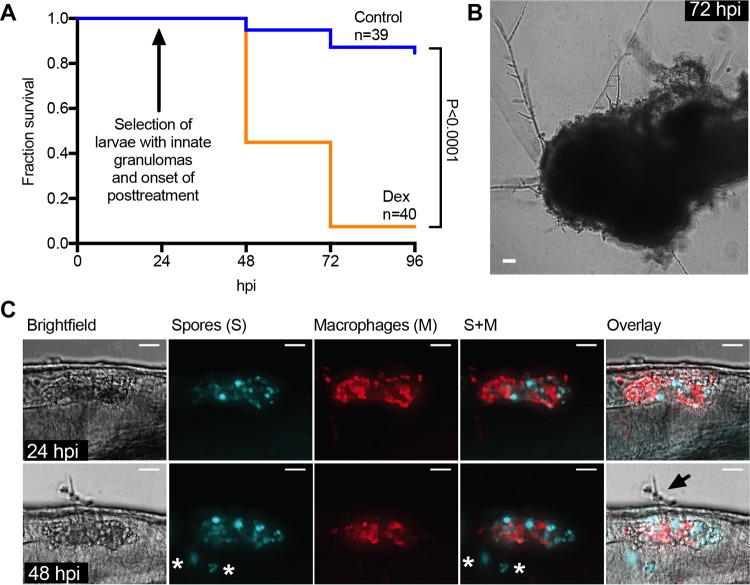
*Mucor circinelloides* can be activated out of innate granulomas by immunosuppression *in vivo*. (A) To investigate the potential of disease activation out of innate granulomas, we selected innate granuloma-positive larvae and immersed them in dexamethasone-containing culture medium to induce immune suppression. While control larvae showed 15.4% mortality, 92.5% of larvae transferred into dexamethasone-containing medium died over the time course of this experiment. (B) Representative image showing that larvae that had succumbed to infection presented with extensive hyphal growth (scale bar, 100 μm). (C) Further microscopic examination revealed that dexamethasone resulted in reduction in innate granuloma size and onset of hyphal growth. The top panels show a representative innate granuloma in the hindbrain ventricle at 24 hpi (before onset of dexamethasone treatment) (maximum-intensity projection of fluorescence z-stack with 19 sections every 4 μm together with bright-field image and overlay, with spores [S] in cyan and macrophages [M] in red; scale bar, 100 μm). The bottom panels show the same innate granuloma at 48 hpi (24 h post-onset of dexamethasone treatment) showing hyphal formation (arrow), reduction in innate granuloma size, and dissemination of spores (asterisks) (maximum-intensity projection of fluorescence z-stack with 36 sections every 4 μm together with bright-field image and overlay, with spores in cyan and macrophages in red; scale bar, 100 μm).

Our *in vivo* spore killing and ROS data suggest that innate granulomas control, but do not clear, fungal spores. To test our hypothesis that latent fungal infections are controlled, but not cleared, by innate granulomas *in vivo*, we reactivated fungal disease by immunosuppression with dexamethasone after formation of innate granulomas. Zebrafish larvae were injected with spores and screened for innate granuloma formation 24 hpi. Innate granuloma-positive larvae were then submerged in dexamethasone-containing growth medium and monitored for survival and by microscopy. Immunosuppression led to rapid mortality that was significantly higher (92.5%) than that in untreated, infected larvae (15.4%) (Fig. 7A) (*P* < 0.0001, Mantel-Cox log-rank test). It is noteworthy that control larvae exhibiting innate granuloma formation also showed a reduced mortality compared to nonselected untreated larvae (which included larvae with and without granulomas), which had an overall mortality of 36.1% (Fig. 2A) (*P* = 0.01, Mantel-Cox log-rank test). This suggests some variability within the larval population that might lead to increased susceptibility in some individuals. Microscopic examination revealed that within as little as 3 h of treatment, innate granulomas failed to suppress spore germination and filamentous growth (Fig. 7B), leading to complete fungal invasion and consumption of the larvae after 48 h (Fig. 7C).

Together, these data support a model in which initial recruitment of phagocytic cells to the site of infection supports granuloma formation, thereby ensuring high local phagocyte number (*K*_*M*_, *K*_*N*_). Innate granulomas are sufficient to control, but not clear, the infection, and loss in maximum phagocyte number either prior to infection or after granuloma formation leads to fulminant mucormycosis and mortality.

## DISCUSSION

We here present a study investigating the temporal-spatial pattern of innate immunity during mucormycosis. We model the early events of innate immune recruitment in response to *M. circinelloides* spores by a combined real-time *in vivo* microscopy and *in silico* modeling approach. In view of our data, we propose that infection with mucormycetes is controlled by innate granulomas, while at the same time inducing a latent stage of infection during mucormycosis that can be reactivated by immunosuppression. This is an expansion of our initial observation that phagocytes accumulate at the site of infection and that failure to do so correlates with spores being disseminated from the site of infection. Specifically, we provide a detailed timeline for innate granuloma formation and show that immunosuppression with dexamethasone reduces innate granuloma formation. We also give insights into how spores maintain latency within innate granulomas by reducing ROS and proinflammatory signals. Lastly, we demonstrate that infection can be activated from a latent stage.

Granulomatous structures have been described in histopathological examinations for a large spectrum of opportunistic fungal infections (e.g., cryptococcosis, mucormycosis, candidiasis, and chromoblastomycosis) (28–30). However, granulomas are best studied in the context of infections with *Mycobacterium tuberculosis*, the causative agent of tuberculosis (TB). Macrophages and neutrophils can control TB by slowing down bacterial growth during early infection, yet also function as vehicles for later tissue dissemination (31, 32). Granuloma formation during TB may provide both a means to control infection and also a niche for bacteria to survive, waiting for an opportunity to break out and cause disease. It is well documented that several fungal pathogens can survive within individual macrophages or neutrophils (e.g., *Cryptococcus neoformans* [33], *Candida albicans* [34], and mucormycetes [35]) (Fig. 6C), dissemination in phagocytes as trafficking vehicles has been proposed for *Cryptococcus neoformans* (36), and immune-inhibitory effects of infectious mucormycete spores on the innate immune system have been reported (11, 12). To survive within a healthy host, pathogens need to overcome the phagocytic antimicrobial machinery. It is currently not clear whether granuloma forms due to failure to kill a pathogen (host induced) or whether failure to kill the bacteria is a result of granuloma formation and thus might be pathogen induced. While innate granuloma formation during infections with opportunistic *M. circinelloides* spores appears to have a protective role for the host by controlling infectious particles, granulomas might also offer a protective niche for delayed disease activation (Fig. 7). Our zebrafish infection model offers an ideal system to fully investigate this.

Mucormycetes are emerging pathogens, with extremely high mortality rates of over 95% in those with disseminated disease (37, 38). This is due to an increase in susceptible individuals, a current lack of therapeutic antifungal interventions, and a current lack of understanding of mucormycosis pathogenesis. Mucormycosis is a suite of diseases caused by a range of opportunistic pathogens, which indicates that healthy individuals can effectively control the infection. Patients that present with mucormycosis commonly show impaired function of effector cells of the innate immune system: i.e., macrophages and neutrophils (1, 5, 9, 10). Thus, an improved understanding of the mechanisms that enable fungal control in healthy individuals will expand our understanding of mucormycosis in susceptible patients and may inform treatment strategies.

In this study, we dissected the *in vivo* real-time dynamics of the innate immune response (Fig. 1) in a zebrafish larval model of mucormycosis (17). We demonstrate that zebrafish larvae initiate a global innate response to fungal spores by increasing their phagocyte population (Fig. 1B and C) and rapidly recruit phagocytes to the site of infection (Fig. 1D and E). This robust early phagocyte recruitment to the site of infection provides protection from disease onset (Fig. 2). *In silico* mathematical modeling enabled us to dissect the parameters of the recruitment response conferring protection. Interestingly, protection appears to be dependent on the number of phagocytes (carrying capacity) at the infection site and less so on the speed of recruitment (Fig. 3). These data expand on previous reports showing that in healthy hosts, phagocytes are recruited to the site of infection and internalize spores in vertebrate models (4, 17, 39–42), thus offering a mechanistic explanation of how this response is disrupted in susceptible hosts (15, 17, 40) (Fig. 2).

Our computational modeling emphasized the importance of macrophage number on disease outcome and predicts which proinflammatory responses may be lacking in zebrafish who do not survive mucormycosis infection (Fig. 5). Ultimately, combining such *in silico* modeling with experimental work should accelerate progress toward the development of therapies to tackle mucormycosis and related infections. Importantly, our data highlight the critical role of prophylactic or early infection control, presenting a major challenge to controlling mucormycosis in the clinic. One therapeutic option would be to support the recruitment of macrophages (*r*_*M*_) and neutrophils (*r*_*N*_) during the initial phases of infection. While infection may be difficult to predict in a clinical setting, the early administration times required in our model simulations for protection against infection are relative to the predicted time of death in the zebrafish larvae, which when untreated, is around 40 h (Fig. 5B). In patients, mortality occurs within a much more variable time frame ranging from days to months, providing a longer working window for successful treatment.

In a clinical setting, a treatment that can be successfully delivered even in the late stages of an infection is most desirable. While phagocytes fail to kill spores (Fig. 5C), they can prevent the germination of spores *in vivo* (15, 18, 43). In susceptible hosts, inhibition of spore germination fails, leading to filamentous growth (15, 18). Our mathematical model further highlights the importance of inhibiting spore germination for protection from active fungal disease. Inhibition of hyphal formation either directly or via immune cell recruitment proved efficacious and may be a plausible control method within a helpful time period (Fig. 6B). Combinations of proinflammatory responses may be capable of facilitating survival in even severely immunocompromised individuals (Fig. 6).

We demonstrate *in vivo* lack of mucormycete spore killing and lack of reactive oxygen burst, which together potentially result in latent fungal infection (Fig. 5C and D). During this latent stage of infection, spores are controlled in innate granulomas *in vivo* and disease can be reactivated by immunosuppression. Unlike TB and other diseases classically associated with granuloma formation, mucormycosis is not associated with T-cell deficiencies, indicating that adaptive immunity has a secondary role in the immune response to mucormycete infection (44). Our model host does not have a developed adaptive immune system and thus relies on innate immune effectors to respond to infectious stimuli, allowing specific dissection of the innate immune response to infections. However, we note that T-cell responses are activated in the context of human disease (45, 46) and might be involved in spore clearance.

Taken together, this is the first real-time analysis of innate granuloma formation during the early stages of a fungal infection. We have presented a novel interdisciplinary strategy to understanding the interplay between the host immune response and mucormycetes. Recent work has shown that spores induce proinflammatory responses upon encountering macrophages (17, 47). Our data suggested that proinflammatory signals are beneficial in controlling mucormycosis by inducing phagocyte recruitment, and both phagocyte number and activity were found to be required for protection from disease onset. While direct phagocyte-mediated fungal killing would be promising as a protective measure from mucormycosis *in silico*, our experimental results showed that phagocytes fail to mount an effective antimicrobial response to spores. We observed a lack of reactive oxygen burst and failure to kill fungal spores *in vivo*. Instead, protection is mediated by robust phagocyte recruitment by innate immune effectors, which drive the formation of early innate granulomas. While spores are successfully contained in these early granulomas (innate granulomas), disease can be reactivated by dexamethasone-induced immunosuppression, indicating the potential for a latent infectious stage during mucormycosis that needs to be considered for clinical management of patients. While frequently observed during fungal infections, the role of granulomas in fungal disease pathogenesis and latency has been overlooked to date. A better understanding of the role of fungal granulomas in latency during fungal infections has the potential to significantly improve our clinical management of patients by enabling the identification of individuals at risk of disease and thus informing the need for prophylactic strategies either directly by targeting the fungal pathogen or indirectly by immunomodulatory interventions.

## MATERIALS AND METHODS

### *Mucor circinelloides* strain and growth conditions.

NRRL3631, a clinical isolate of *Mucor circinelloides*, was used in this study (35). The fungus was grown at a density of 1,000 spores per 10-cm agar plate on Sabouraud dextrose (SAB) agar (pH 5.6) containing 0.5% (wt/vol) peptone from casein, 0.5% (wt/vol) peptone from meat, 4% (wt/vol) d(+)-glucose and 1.5% (wt/vol) agar (Merck Millipore, Watford, United Kingdom) in the dark at room temperature (17).

### Zebrafish care and maintenance.

Adult zebrafish were kept in recirculating systems (ZebTec Active Blue zebrafish housing system; Tecniplast, London, United Kingdom) at the University of Birmingham’s zebrafish facility under a 14- to 10-h light-dark cycle and water temperature maintained at 28°C. All zebrafish care and experiments were conducted according to Home Office legislation and the Animals (Scientific Procedures) Act 1986 (ASPA) under Home Office project license 40/3681 and personal licenses l13220C2C to Kerstin Voelz and l9EE992EF to Aleksandra Jasiulewicz. Zebrafish eggs were obtained by natural mating. Eggs were collected, and 25 eggs were kept in 25 ml of E3 medium plus 0.00003% methylene blue (Sigma-Aldrich, Irvine, United Kingdom) and 26.6 μg/ml phenylthiourea (PTU; Sigma-Aldrich, Irvine, United Kingdom) at 32°C and under a 14- to 10-h light-dark cycle thereafter. Medium was changed every 2 days up to 5 days postfertilization (dpf). This study utilized wild-type AB zebrafish as well as transgenic zebrafish Tg(mpx:GFP)^i114^ expressing green fluorescent protein in neutrophils (48), Tg(mpeg1:Gal4-FF)^gl25^ (49) crossed with Tg(UAS-E1b:NfsB.mCherry)^c264^ [herein referred to as Tg(mpeg1:G/U:NfsB-mCherry)] with macrophage-specific expression of red fluorescent protein mCherry and nitroreductase, and Tg(mpeg1:G/U:NfsB-mCherry) crossed with Tg(mpx:GFP) [herein referred to as Tg(mpeg1:G/U:NfsB-mCherry/mpx:GFP)] with neutrophil-specific expression of green fluorescent protein and macrophage-specific expression of red fluorescent protein mCherry. All zebrafish care and husbandry procedures were performed as previously described, and larvae were observed throughout the experimental procedures for normal anatomical development (17, 50).

### Spore preparation for injection.

Prior to injections, sporangiospores were collected by gentle washing of the mycelium with 10 ml of Dulbecco’s phosphate-buffered saline (PBS) (Sigma-Aldrich, Irvine, United Kingdom). The spore suspension was washed with PBS, and spore numbers were enumerated using a hemocytometer. For visualization purposes, 10^8^ spores were stained with 100 μg/ml Fluorescent brightener 28 (Sigma-Aldrich, Irvine, United Kingdom), washed with PBS, and resuspended in 1 ml of 10% polyvinylpyrrolidone (PVP) (40 kDa; Sigma-Aldrich, Irvine, United Kingdom) with 0.05% phenol red as an injection marker as previously described (17).

### Hindbrain ventricle injection.

Hindbrain injections were conducted as previously described (17, 51). Briefly, the developmental stage of zebrafish embryos was assessed according to Kimmel et al. for injection at the prim-25 stage (52). Embryos were manually dechorionated and anesthetized with 160 μg/ml ethyl 3-aminobenzoate methanesulfonate salt (tricaine; Sigma-Aldrich, Irvine, United Kingdom). Transgenic embryos were screened and selected for expression of the cell-specific fluorescent label prior to injection. Infection doses of 100 sporangiospores per fish were achieved by microinjecting 2 nl of a 10^8^-spore/ml suspension in 10% PVP through the otic vesicle into the hindbrain ventricle of the zebrafish embryo; 2 nl of PVP was injected as a control. The carrier medium PVP was chosen due to its increased density, enabling increased injection consistency and reduced clogging of microinjection needles. Infection dose and location were confirmed microscopically immediately after microinjection. Larvae were kept in 200 μl of E3 in individual wells of 96-well plates with or without chemical manipulation. Larvae were monitored every 24 h for phagocyte numbers/recruitment, granuloma formation, hyphal growth, dissemination, and spore and larval survival up to 5 dpf. At 5 dpf, larvae were sacrificed with a tricaine overdose (10×).

### Chemical treatments.

Immune suppression was mimicked by treatment with the steroid drug dexamethasone (Dex) (14, 17). Larvae were immersed in E3 with 40 μg/ml water-soluble dexamethasone (Sigma-Aldrich, Irvine, United Kingdom) 4 h prior to microinjection of spores and maintained in dexamethasone-containing E3 for survival and phagocyte recruitment studies. For reactivation studies, larvae were transferred from E3 to E3 containing dexamethasone after formation of innate granulomas.

### Spore viability assessment.

Spore viability was assessed as previously described (17). Briefly, after microinjection of fungal spores, larvae were individually euthanized with a 10× overdose of tricaine and homogenized in 100 μl penicillin-streptomycin (10,000 U/ml penicillin and 10 mg/ml streptomycin; Sigma-Aldrich, Irvine, United Kingdom) and plated onto SAB agar plates with 100 U/ml penicillin, 100 μg/ml streptomycin, and 30 μg/ml gentamicin (Sigma-Aldrich, Irvine, United Kingdom). Plates were incubated at room temperature for 24 to 48 h before CFU were counted.

### Fluorescence microscopy.

For microscopic analysis, individually kept zebrafish embryos were anesthetized with 200 μg/ml tricaine in E3 in 96-well plates. Phagocyte numbers at the site of infection and in the whole embryos, innate granuloma formation, spore dissemination, and fungal filamentous growth within infected embryos was assessed through a Zeiss Axio observer Z1 microscope with an ApoTome system (Zeiss, Cambridge, United Kingdom) using objective lenses with powers of 10×/0.25 numerical aperture (NA), 20×/0.4 NA, and 40×/0.6 NA (Zeiss, Cambridge, United Kingdom). Green fluorescent protein, mCherry, and fluorescent brightener fluorescent signals were detected by their emission signals at 509 nm (excitation, 488 nm), 610 nm (excitation, 587 nm), and 465 nm (excitation, 353 nm), respectively. Images were taken and processed with the open community platform for BioImage informatics Icy (53), and figures were compiled in Illustrator C3 (Adobe Systems, San Jose, CA, United States). For cell tracking analysis, movies were analyzed using Volocity software (Improvision, Coventry, United Kingdom) to determine the mean fluorescence intensity of mCherry (macrophages) or GFP (neutrophils) across the field of view at each time point over the 12-h imaging period, and data were normalized to the first frame. Innate granuloma formation was defined as accumulation of >10 phagocytes in a 40- by 40-μm area. A spore dissemination event was recorded if there was at least one spore that was observed outside the hindbrain ventricle compartment (17).

### Measurement of reactive oxygen species production.

In order to evaluate the reactive oxygen burst induced in response to infection with fungal spores, the oxidative stress indicator chloromethyl derivative 5-(and-6)-chloromethyl-2′,7′-dichlorohydrofluorescein diacetate (CM-H_2_DCFDA; Invitrogen, Fisher Scientific, Loughborough, United Kingdom) was used to measure reactive oxygen species (ROS). Infected larvae were incubated in pairs in E3 containing 5 mM of CM-H_2_DCFDA in 96-well plates, and ROS production was measured as fluorescence from CM-H_2_DCFDA upon oxidation by ROS. Fluorescence was measured at 520 nm (excitation, 485 to 12 nm) with the FLUOstar Omega microplate reader (BMG Labtech, Aylesbury, United Kingdom). One hundred nanograms per milliliter phorbol 12-myristate 13-acetate (PMA; Sigma-Aldrich, Irvine, United Kingdom) was used as a positive control. Wells with dead fish were excluded from the analysis.

### qRT-PCR.

One hour after injections of PVP or fungal spores into control or dexamethasone-treated larvae, 15 fish were placed in Eppendorf tubes, euthanized with an overdose of tricaine, and homogenized. Total RNA was isolated from homogenates using the PureLink RNA minikit (Thermo, Fisher Scientific) according to the manufacturer’s instructions and reverse transcribed into cDNA using the using iScript cDNA synthesis kit (Bio-Rad). Quantitative reverse transcription-PCR (qRT-PCR) was performed using the Brilliant III Ultra-Fast SYBR green (Agilent) for *arp* (housekeeping gene) with forward primer 5′ CTGCAAAGATGCCCAGGGA 3′ and reverse primer 5′ TTGGAGCCGACATTGTCTGC 3′, *tnf*-α with forward primer 5′ TCGCATTTCACAAGGCAATTT 3′ and reverse primer 5′ GGCCTGGTCCTGGTCATCTC 3′, and *il-8* with forward primer 5′ GTCGCTGCATTGAAACAGAA 3′ and reverse primer 5′ CTTAACCCATGGAGCAGAGG 3′ (Eurofins Scientific). Amplification was performed with the Aria Mx (Agilent) thermocycler and the following conditions: 10 min of denaturation at 95°C, followed by 40 cycles of 15 s at 95°C and 1 min at 60°C. A final dissociation curve was performed for each group. Threshold cycles (*C*_*T*_) and dissociation curves were analyzed with the Aria MX software package. Gene expression levels were normalized to zebrafish *arp* (Δ*C*_*T*_) and compared with PVP-injected controls (ΔΔ*C*_*T*_). Results are presented as fold change (2^ΔΔ*CT*^) (17, 54).

### Data analysis and statistics.

Data were collected from at least three independent experimental repeats. Exact numbers of experimental repeats and embryos investigated are given where indicated in the Results section. Data were analyzed with GraphPad Prism version 7.0a software (GraphPad, La Jolla, CA). The statistical analysis methods for the different data sets are specified in the Results section and figure legends. *P* values of <0.05 were considered statistically significant and are indicated in the figures as follows: *, *P* < 0.05; **, *P* < 0.01; and ***, *P* < 0.001.

### Mathematical model formulation.

We developed an ordinary differential equation (ODE) model of the early stages of a mucormycosis infection to explore immunomodulation effects. Related ODE models of immunomodulation include applications to bacterial infections (55), HIV (56), and oncolysis (57), while an agent-based model is used to explore diverse movement strategies of neutrophils toward fungal spores in the study by Tokarski et al. (58).

Our mucormycosis infection model consists of four interacting components (Fig. 3A and B): the population (at the site of infection) of infectious fungal spores (*S*), the hyphae produced by spore germination (*H*), and the available pools of macrophages (*M*) and neutrophils (*N*). (Both are recruited to the infection via the presence of spores and inhibit formation of hyphae.) In brief, we use mass action kinetics, including saturation terms where necessary (in particular we square the numbers of macrophages and neutrophils on the denominator of equations 3 and 4 from panel B following a model selection process in which this was found to improve the model fit to the data), making the assumption that the system is well mixed and spatial dependencies can be ignored. To generate the model, events were assumed to occur as described below. Infectious spores initiate events and form hyphae at rate *r*_*H*_. Macrophages are recruited to the infection site through the presence of spores at rate *r*_*M*_. Based on results that neutrophils are delayed from arriving at the site of infection relative to macrophages (see Results and Fig. 1D and E), neutrophils are assumed to be recruited in response to the presence of macrophages at rate *r*_*N*_. At the site of infection, we assume that there are maximum numbers of macrophages and neutrophils (*K*_*M*_ and *K*_*N*_, respectively) that are recruited (akin to the carrying capacity widely used in modeling in microbiology). *K*_*M*_ and *K*_*N*_ are fixed parameters that we vary between simulations to mimic zebrafish with different immune response strengths. Macrophages and neutrophils act independently to inhibit hyphae formation described by the rates β_*M*_ and β_*N*_, respectively, but they do not kill *Mucorales* (see Results and Fig. 5A). Death of immune cells is negligible over the time course of interest. We note that we have investigated the possibility that hyphae also attract macrophages to the infection site, but as hyphae formation is generally established as the phagocytes are approaching carrying capacity, this had no qualitative effect on our results; we therefore omit this possibility for simplicity.

### Mathematical model parameterization.

Initial numbers of macrophages, neutrophils, and hyphae at the site of the infection were set to 0 based on *in vivo* observations, while the initial number of spores was 100 (as per the experimental protocol). Parameterization was performed by minimizing an appropriate objective function using *fminsearch* in MATLAB. Estimates of the recruitment rates *r*_*M*_ and *r*_*N*_ were based on the time series raw data of the recruitment *in vivo* assays (see Results and Fig. 1D and E; with objective function the sum of the *l*^2^-norm of the model errors for macrophage and neutrophil count using a reduced model for phagocyte recruitment only). Note that though the units are different between these data and the variables in the model (and there is currently no obvious means to convert between the two), we have used the data to obtain information about the relative sizes of these two parameters. To address this uncertainty, these (and indeed all) parameters are subject to sensitivity analyses performed later in the study.

*K*_*M*_ and *K*_*N*_ are assumed to vary between zebrafish and were taken as the number of macrophages and neutrophils, respectively, at the infection site after 24 h (see Table S1 in the supplemental material) as experimental observations indicate phagocyte numbers have saturated by this time point. The full model was simulated against these data, and the objective function was used to estimate the remaining parameters. The objective function is given by the *l*^2^-norm of the vector, *v*_*i*_, where for *i* = 1 … 16 (deceased fish [Table S1A])

$$v_{i}=\{\begin{array}{ll} 0 & \text{if }t_{i}^{s}\in (t_{i}^{*}-24,\;t_{i}^{*}],\\ (t_{i}^{*}-24)-t_{i}^{s} & \text{if }t_{i}^{s}<t_{i}^{*}-24,\\ t_{i}^{s}-t_{i}^{*} & \text{if }t_{i}^{s}>t_{i}^{*} \end{array}$$

with $t_{i}^{s}$ and $t_{i}^{*}$, the simulated and recorded death times, respectively. (Note that a fish may have died at any point in the 24 h preceding the recorded death time.) For *i* = 17 … 36 (fish surviving to 120 h [Table S1B]), $v_{i}=\max(120-t_{i}^{s},\;0)$.

10.1128/mBio.02010-17.4 TABLE S1  Phagocyte recruitment and larval survival after infection with *M. circinelloides* spores *in vivo*. Download TABLE S1, DOCX file, 0.1 MB. Copyright © 2018 Inglesfield et al. 2018 Inglesfield et al. This content is distributed under the terms of the Creative Commons Attribution 4.0 International license.

To calculate a simulated time of death, it is assumed that hyphae amass until they reach a critical value, termed H*, at which point host damage is correspondent with death. (H* must also be estimated as part of the parameterization process.) Latin hypercube sampling was used to generate a range of starting guesses (*fminsearch* is a local optimizer). We subsequently explored a range of promising parameter sets that gave equivalent qualitative results throughout. We present only one of these here (that which gave the lowest obtained objective function value): *r*_*M*_ = 0.013 (h^−1^), *r*_*N*_ = 0.033 (h^−1^), *r*_*H*_ = 2.32 (h^−1^), β_*M*_ = 1.30 (fungal cells)(immune cells)^−1^, β_*N*_ = 1.02 (fungal cells)(immune cells)^−1^, H* = 58.85 (fungal cells), and *K*_*H*_ = 1.12 (fungal cells), with *K*_*N*_ and *K*_*M*_ taken for each zebrafish from Table S1A and B. Results of this parameterization are given in Fig. S3A and B in the supplemental material. We note that where required fold changes in parameters for specific outputs are stated in Results, these are given for comparison purposes between cases investigated within one parameter set and may differ for alternative parameter sets (while remaining qualitatively equivalent).

10.1128/mBio.02010-17.3 FIG S3  Mathematical model fit to the recruitment/survival data given in Table S1. Solid lines represent spore number during the course of infection, while hyphae are given by the dashed lines. Each panel represents a zebrafish from (A) those that died before 120 h in Table S1A and (B) those that survived until 120 h in Table S1B. (Different macrophage and neutrophil carrying capacities are inputted to the model simulation for individual zebrafish, as given by the values at 24 h.) (A) The asterisk denotes the time at which the fish death was recorded and the triangle 24 h preceding this. (The fish may have died at any time in that interval.) The height of these two symbols corresponds to the critical level of hyphae, at which it is assumed that the fish die (H*). The model correctly predicts the time of death, therefore, if the dashed line passes between the two symbols. Under the default parameter regime (see Materials and Methods), the model predicts death during a correct interval in all but six cases. (B) The dotted line corresponds to the critical level of hyphae at which it is assumed that the fish die (H*). The model correctly predicts the time of death, therefore, if the dashed line does not cross the dotted line before 120 h. Under the default parameter regime (see Materials and Methods), the model correctly predicts survival in all but five cases. Download FIG S3, PDF file, 0.1 MB. Copyright © 2018 Inglesfield et al. 2018 Inglesfield et al. This content is distributed under the terms of the Creative Commons Attribution 4.0 International license.

### Ethics statement.

All zebrafish care and experiments were conducted according to Home Office legislation and the Animals (Scientific Procedures) Act 1986 (ASPA) under Home Office project license 40/3681 and personal licenses l13220C2C to Kerstin Voelz and l9EE992EF to Aleksandra Jasiulewicz.
