# Supplementary figures and images for: Robust Phagocyte Recruitment Controls the Opportunistic Fungal Pathogen Mucor circinelloides in Innate Granulomas In Vivo
Source: mBio. 2018 Mar 27;9(2):e02010-17. doi: 10.1128/mBio.02010-17 (PMC5874920; doi:10.1128/mBio.02010-17)

**A**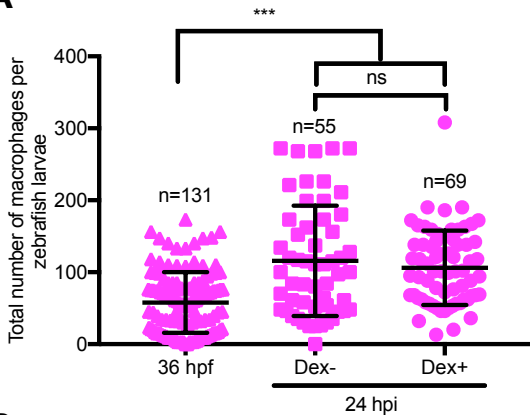**B**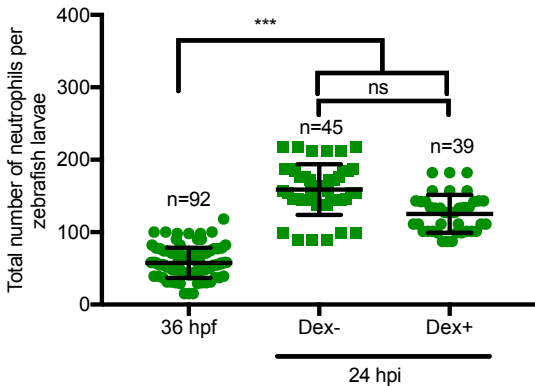

Supplement: FIG S1 [file mbo002183792sf1.pdf]

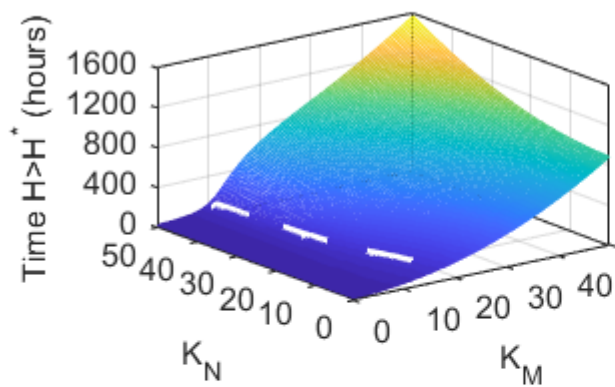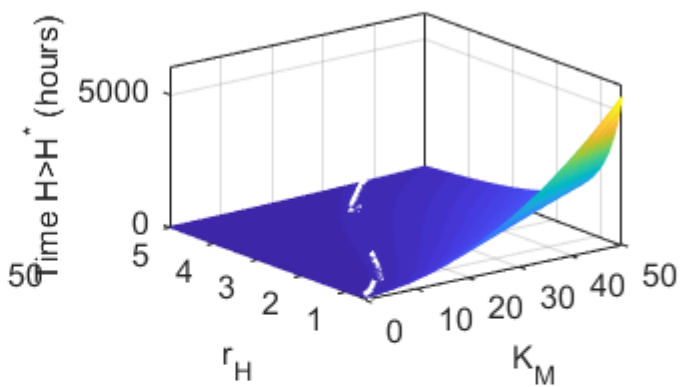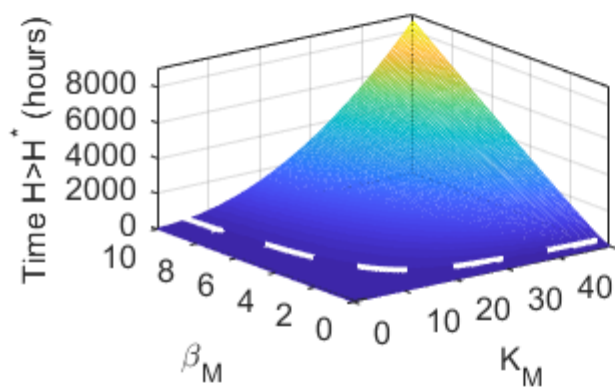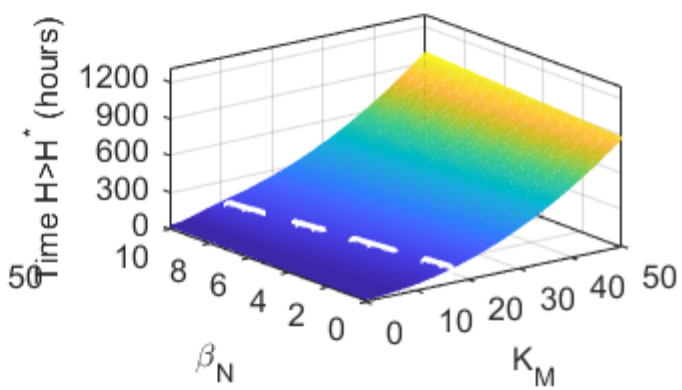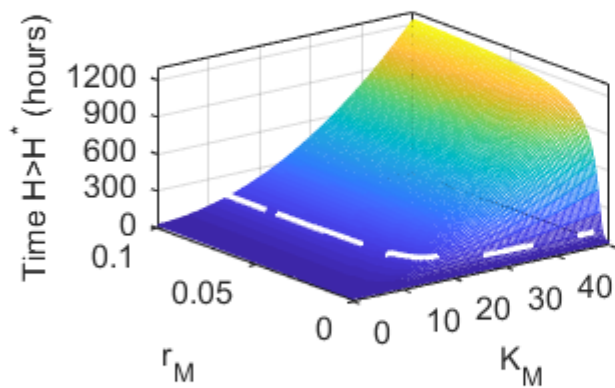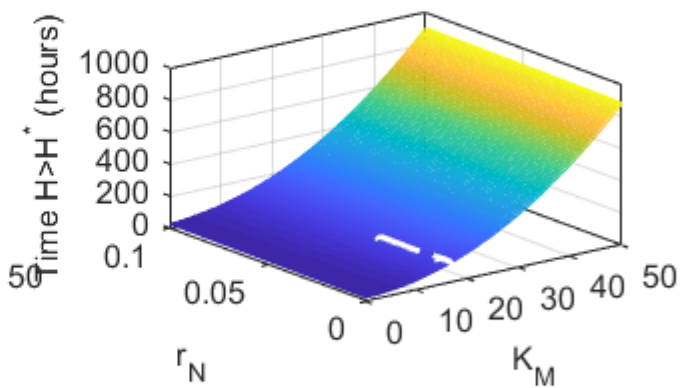

Supplement: FIG S2 [file mbo002183792sf2.pdf]

**A**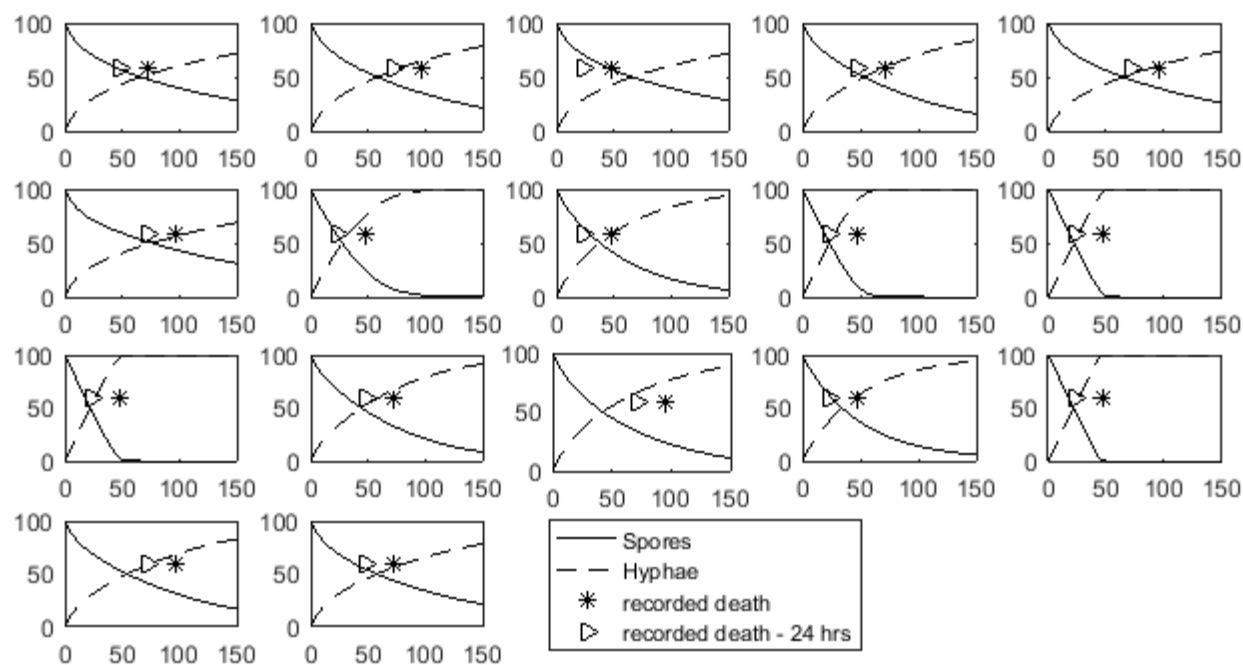**B**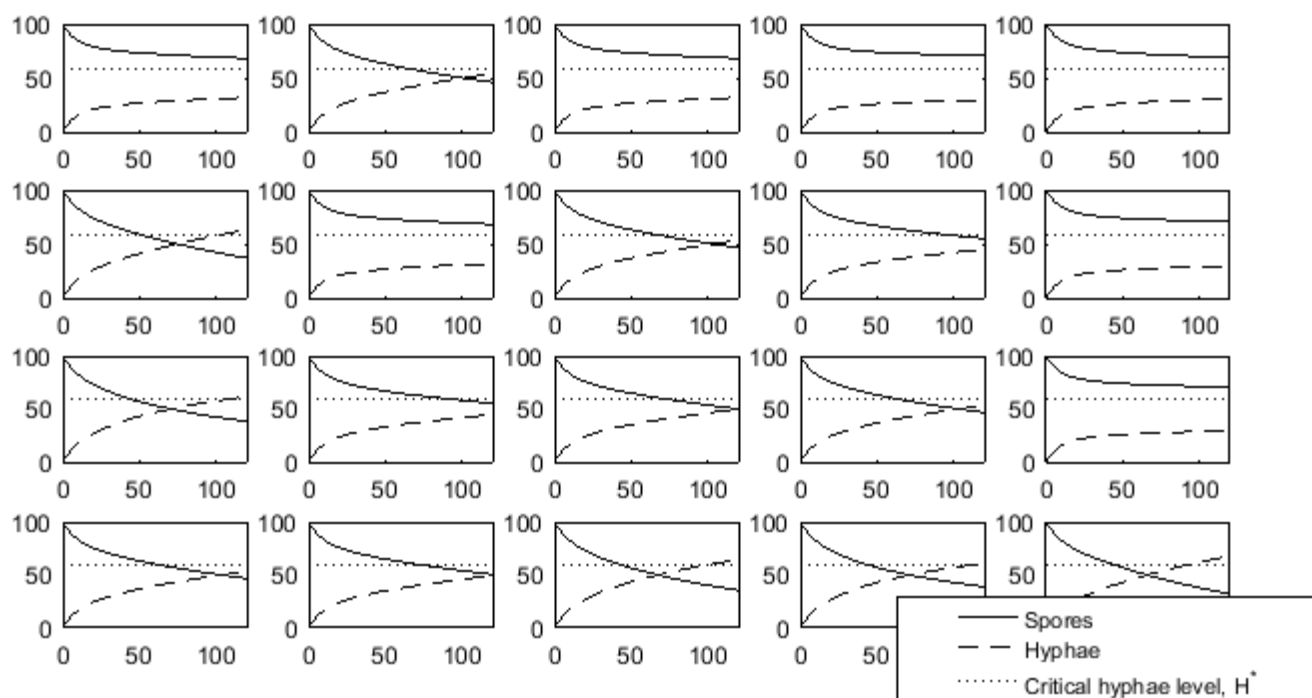

Supplement: FIG S3 [file mbo002183792sf3.pdf]
