# Supplementary material for: Robust Phagocyte Recruitment Controls the Opportunistic Fungal Pathogen Mucor circinelloides in Innate Granulomas In Vivo
Source: mBio. 2018 Mar 27;9(2):e02010-17. doi: 10.1128/mBio.02010-17 (PMC5874920; doi:10.1128/mBio.02010-17)
Supplement: TABLE S1 [file mbo002183792st1.docx]

**Supplementary Table**

**Table S1: Phagocyte recruitment and larval survival after infection with *M. circinelloides* spores *in vivo*.** Zebrafish larvae with fluorescently labelled macrophages (mCherry) and neutrophils (GFP) were microinjected with approximately 100 spores and phagocyte recruitment assessed after 24 hours. Individual larvae were then followed until death **(A)** or the limit of the experimental time frame (96 hours). Larvae still alive at this timepoint were assigned a survival of 120 hours **(B)**.

**A**

| Deceased zebrafish | | |
| --- | --- | --- |
| Number of neutrophils in hindbrain at 24 hours | Number of macrophages in hindbrain at 24 hours | Time at which death recorded (hrs) |
| 11 | 12 | 48 |
| 4 | 4 | 48 |
| 7 | 7 | 48 |
| 5 | 2 | 48 |
| 11 | 1 | 48 |
| 9 | 1 | 48 |
| 9 | 6 | 48 |
| 11 | 12 | 72 |
| 3 | 11 | 72 |
| 3 | 9 | 72 |
| 8 | 11 | 72 |
| 8 | 11 | 96 |
| 9 | 12 | 96 |
| 8 | 14 | 96 |
| 8 | 8 | 96 |
| 4 | 11 | 96 |

**B**

| Fish surviving to 120 hours | |
| --- | --- |
| Number of neutrophils in hindbrain at 24 hours | Number of macrophages in hindbrain at 24 hours |
| 11 | 50 |
| 10 | 17 |
| 11 | 50 |
| 50 | 50 |
| 19 | 50 |
| 7 | 14 |
| 12 | 50 |
| 11 | 17 |
| 17 | 21 |
| 50 | 50 |
| 16 | 12 |
| 18 | 21 |
| 11 | 19 |
| 11 | 17 |
| 50 | 50 |
| 11 | 17 |
| 12 | 19 |
| 12 | 12 |
| 17 | 12 |
| 5 | 13 |
